# Behaviour Concerns in Preschool Cardiac Surgery Survivors

**DOI:** 10.1016/j.cjcpc.2024.04.001

**Published:** 2024-04-26

**Authors:** Sabrina H.Y. Eliason, Charlene M.T. Robertson, Susan A. Bobbitt, Sara Khademioureh, Irina A. Dinu, Ari R. Joffe, Bryan V. Acton

**Affiliations:** a Faculty of Medicine & Dentistry, University of Alberta, Edmonton, Alberta, Canada; b Complex Pediatric Therapies Follow-Up Program, Glenrose Rehabilitation Hospital, Edmonton, Alberta, Canada; c Department of Pediatrics, University of Saskatchewan, Saskatoon, Saskatchewan, Canada; d School of Public Health, University of Alberta, Edmonton, Alberta, Canada; e Department of Psychology and Health Studies, University of Saskatchewan, Saskatoon, Saskatchewan, Canada

## Abstract

**Background:**

Behaviour concerns (BC) are reported in survivors of complex cardiac surgery (CCSx) with inconsistent evidence about health and demographic variables that impact outcomes.

**Methods:**

A prospective inception-cohort study of infants (without known chromosomal abnormalities) after CCSx from 2001 to 2017 determined Behaviour Assessment System for Children (BASC-II/III) parent rating scales at 4.5 years. *T* scores ≥60 for externalizing, internalizing, and the Behavioural Symptoms Index and ≤40 for adaptive behaviour defined BC. Potential predictive variables included demographic, acute care, and health factors after initial CCSx. Multiple logistic regression using the purposeful selection method gave odds ratios (ORs) with 95% confidence intervals (CIs).

**Results:**

Survivors (n = 585; 61% boys, 40% single ventricle) were assessed at a median age of 55 months (interquartile range: 53, 57 months). Independent predictors were noncardiac hospitalizations (OR: 1.10, 95% CI: 1.02, 1.19; *P* = 0.015) for externalizing; noncardiac hospitalizations (OR: 1.14, 95% CI: 1.05, 1.24; *P* = 0.003), female sex (OR: 1.62, 95% CI: 1.04, 2.52; *P* = 0.031), and single ventricle (OR: 1.82, 95% CI: 1.04, 3.17; *P* = 0.035) for internalizing; noncardiac hospitalizations (OR: 1.10, 95% CI: 1.02, 1.19; *P* = 0.017), socioeconomic status (SES) (OR: 0.98, 95% CI: 0.96, 0.10; *P* = 0.031), and years of maternal schooling (OR: 0.91, 95% CI: 0.84, 0.10; *P* = 0.04) for adaptive; and extracorporeal life-saving support (OR: 2.03, 95% CI: 1.01, 3.96; *P* = 0.041) for the Behavioural Symptoms Index, indicating more pervasive behaviours.

**Conclusions:**

The number of noncardiac hospitalizations predicted increased odds of BC and requires further attention. Improving inpatient trauma-informed care experiences and optimizing access to primary care to prevent noncardiac hospitalization may be modifiable.

## Graphical abstract

**Figure:**
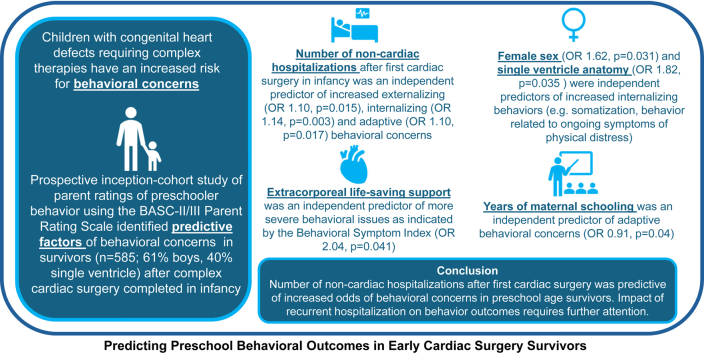

## Résumé

**Contexte:**

Des problèmes de comportement sont signalés chez les enfants ayant subi une intervention chirurgicale cardiaque complexe (ICCC). Or, il existe des incohérences dans les données probantes dont nous disposons sur les variables démographiques ou celles liées à la santé qui peuvent influencer ces résultats.

**Méthodologie:**

Nous avons mené une étude prospective auprès d’une cohorte initiale de nourrissons (sans anomalie chromosomique connue) ayant subi une ICCC entre 2001 et 2017, pour lesquels les résultats au questionnaire du parent du Système d’évaluation du comportement de l’enfant (*Behavior Assessment System for Children*, BASC-2/3) ont été obtenus à l’âge de 4,5 ans. Les problèmes de comportement ont été définis comme l’obtention de notes T ≥ 60 pour les problèmes d’externalisation, les problèmes d’internalisation et l’indice des symptômes comportementaux (ISC) et de ≤ 40 pour les compétences adaptatives. Les variables à valeur prédictive potentielle incluaient des facteurs démographiques et d’autres liés aux soins aigus ou à la santé après une première ICCC. Une analyse par régression logistique multiple avec sélection intentionnelle des variables a permis d’obtenir des rapports de cotes (RC) avec un intervalle de confiance (IC) à 95 %.

**Résultats:**

Les enfants ayant survécu (n = 585; 61 % de garçons, 40 % présentant un cœur univentriculaire) ont été évalués à un âge médian de 55 mois [intervalle interquartile : 53 à 57 mois]. Les facteurs prédictifs indépendants étaient les suivants : les hospitalisations pour des motifs autres que cardiaques (RC : 1,10, IC à 95 % : 1,02 à 1,19, *p* = 0,015) dans le cas des problèmes d’externalisation; les hospitalisations pour des motifs autres que cardiaques (RC : 1,14, IC à 95 % : 1,05 à 1,24, *p* = 0,003), le sexe féminin (RC : 1,62, IC à 95 % : 1,04 à 2,52, *p* = 0,031) et un cœur univentriculaire (RC : 1,82, IC à 95 % : 1,04 à 3,17, *p* = 0,035) dans le cas des comportements d’internalisation; les hospitalisations pour des motifs autres que cardiaques (RC : 1,10, IC à 95 % : 1,02 à 1,19, *p* = 0,017), le statut socioéconomique (RC : 0,98, IC à 95 % : 0,96 à 0,10, *p* = 0,031) et le nombre d’années de scolarité de la mère (RC : 0,91, IC à 95 % : 0,84 à 0,10, *p* = 0,04) dans le cas des comportements adaptatifs; et le recours à des mesures extracorporelles de soutien vital (RC : 2,03, IC à 95 % : 1,01 à 3,96, *p* = 0,041) dans le cas de l’ISC, ce qui indique des comportements à portée plus large.

**Conclusions:**

Le nombre d’hospitalisations pour des motifs autres que cardiaques, qui était un facteur prédictif d’un risque accru de problèmes de comportement, devrait faire l’objet d’autres études. L’amélioration de l’expérience de soins qui tient compte des traumatismes et l’optimisation des soins primaires pour prévenir les hospitalisations pour des motifs non cardiaques sont des leviers qui pourraient être mis à contribution.

Progress in medical and surgical interventions has contributed to increasing survival in children with congenital heart defects (CHD) after complex cardiac surgery (CCSx). This progress has led to increasing interest in factors that can impact long-term neurodevelopmental and behavioural functioning.

Paediatric survivors of life-saving procedures for the treatment of congenital heart disease are at higher risk for functional challenges in a number of domains including communication, motor skills, cognition, attention, executive functioning, and adaptive abilities.^1^, ^2^, ^3^, ^4^, ^5^, ^6^, ^7^, ^8^ Both internalizing and externalizing behaviours in children with CHD have also been described, especially in areas of anxiety and somatic complaints,^5^^,^^7^^,^^9^, ^10^, ^11^ with most relationships being observed in school-age children or adolescents.

Risk factors associated with these behavioural outcomes have been inconsistent in infant and preschool samples.^12^ Factors impacting behavioural presentation have included family functioning, patient factors such as birth weight, the presence or absence of a known genetic syndrome, length of hospitalization, medical complications after discharge, and features of both the cardiac condition and perisurgical variables.^9^^,^^13^^,^^14^ Previous studies have not been prospective inception-cohort studies, restricted to those at highest risk (ie, having CCSx completed in early infancy), or examined the breadth of risk factors for behavioural outcomes that were included in our study. The current study had the potential to add important information to the literature.

The first purpose of this study was to report on behavioural concerns (BC), defined as clinical and at-risk behaviours as determined by the Behaviour Assessment System for Children (BASC), of preschool children with CHD after early CCSx. The second purpose was to determine whether there were predictive factors across acute care, demographic, and health variables that were associated with behavioural outcomes in preschool survivors of CCSx.

## Methods

This prospective inception-cohort outcomes study was part of a longitudinal follow-up project (the Complex Pediatric Therapies Follow-up Program [CPTFP]) conducted in 6 developmental rehabilitation referral sites in Western Canada. These included Vancouver, British Columbia; Edmonton and Calgary, Alberta; Regina and Saskatoon, Saskatchewan; and Winnipeg, Manitoba.^2^ Infants who were appropriate for the CPTFP were identified at the time of first CCSx. These infants were followed prospectively.

Predetermined demographic, operative, perioperative, and postoperative variables were collected. Need for life-saving support was defined as any one of ventricular assistive device, extracorporeal membrane oxygenation, or heart transplantation. Cardiac hospitalizations were prespecified to be those that were due to cardiac failure, catheterization, or cardiac surgery or due to postcardiac surgical complications. These were often anticipated admissions for Glenn or Fontan palliation surgery. All other hospitalizations were considered noncardiac. Known chromosomal abnormality was identified by the tests available at the time and would have included karyotyping and eventually microarray or cardiac panels. All babies followed in the clinic received genetic testing. If further testing such as Fragile X was indicated, it was ordered based on clinician judgement. Children with a previously identified genetic condition were not included in our sample. Total hospitalization and ventilation days included time during the first CCSx and during all cardiac hospitalizations up to the age of 4.5 years.

### Participants

Inclusion criteria were infants requiring CCSx (those ≤6 weeks of age with CHD and who were considered at greatest risk for adverse outcomes because of the need for CCSx requiring cardiopulmonary bypass, those ≤6 months having cardiac shunt surgery including Glenn surgery, and those <12 months having surgery for isomerisms) and surgery performed from January 2001 to December 2017 at the Stollery Children’s Hospital in Edmonton, Alberta, Canada. Exclusion criteria were death before the 4.5-year follow-up assessment and known chromosomal abnormality. Loss to follow-up was recorded, and those survivors who had incomplete assessments, had parents who refused or were unable to complete questionnaires, missed completion of the BASC because of virtual or partial assessments during the COVID-19 pandemic, were too ill, or were lost to the CPTFP were included. Parents of children who were non-English speaking were supported by a translator in clinic to complete questionnaires.

### Childhood clinical assessments

Multidisciplinary developmental assessments were performed at 4.5 years at the referral sites.^2^ The BASC-II or -III^15^^,^^16^ was completed by the parents of children attending appointments for their developmental follow-up.

### Measures

The BASC-II and -III provide clinical scale and composite scores that provide normative ratings of children’s emotions and behaviour. The BASC can be used in individuals between 2 and 25 years of age. The BASC parent rating scales were used at age 4.5 years in our study. The general norms based on age and sex were used to derive scores.

Composite scores in the BASC include internalizing, externalizing, the Behavioural Symptoms Index (BSI), and adaptive behaviour. Internalizing scores are based on the anxiety, depression, and somatization scales. Externalizing scores are based on the aggression and hyperactivity scales. Adaptive scores are based on the functional communication, adaptability, social skills, and activities of daily living scales. Finally, the BSI is based on results from the hyperactivity, aggression, depression, attention problems, atypicality, and withdrawal scales. Clinical scale and composite scores are represented as *T* scores with a mean and standard deviation of 50.^10^ For externalizing and internalizing behaviour and the BSI, *T* scores at or above 70 indicate “clinically significant” behaviours and *T* scores between 60 and 69 indicate “at-risk” behaviours. For adaptive behaviours, *T* scores below 30 indicate “clinically significant” behaviours and *T* scores between 30 and 39 indicate “at-risk” behaviours. *T* scores ≥60 for externalizing, internalizing, and the BSI, and ≤40 for adaptive behaviour defined clinically relevant BC. This cutoff definition would identify families who self-identified concerns in their child and would potentially be receptive of intervention or further counselling.

### Descriptive variables

Descriptive information about the population studied included a socioeconomic index;^17^ mother’s schooling (years); gestational age (weeks); birth weight (g); sex; antenatal diagnosis; surgical severity, complexity, and major complications until the 4.5-year assessment (including single ventricle, total cardiopulmonary bypass time in minutes, convulsions, dialysis, cardiopulmonary resuscitation, sepsis,^18^ extracorporeal membrane oxygenation, ventricular assist device, and heart transplantation); total hospitalization (days); and total ventilation time (days).

### Statistical analysis

Continuous variables are presented as mean (standard deviation) or median (interquartile range), and categorical variables are presented as counts and percentages. The frequency of the behavioural presentation is provided as a percentage of assessed survivors using 95% confidence intervals (CIs). Demographic, operative, and perioperative predictors of behavioural presentations were analyzed using purposeful selection multiple logistic regression analysis. Multiple logistic regression analysis included variables significant at *P* < 0.10 from univariate analysis as defined by *T* scores ≥60 for externalizing, internalizing, and the BSI, and ≤40 for adaptive behaviour after screening for multicollinearity. Results were expressed as odds ratios (ORs) with 95% CIs. Significance was considered *P* < 0.05. Data analyses also used Levene’s test for quality of variances and the *t* test for equality of means.

## Results

**Figure 1:**
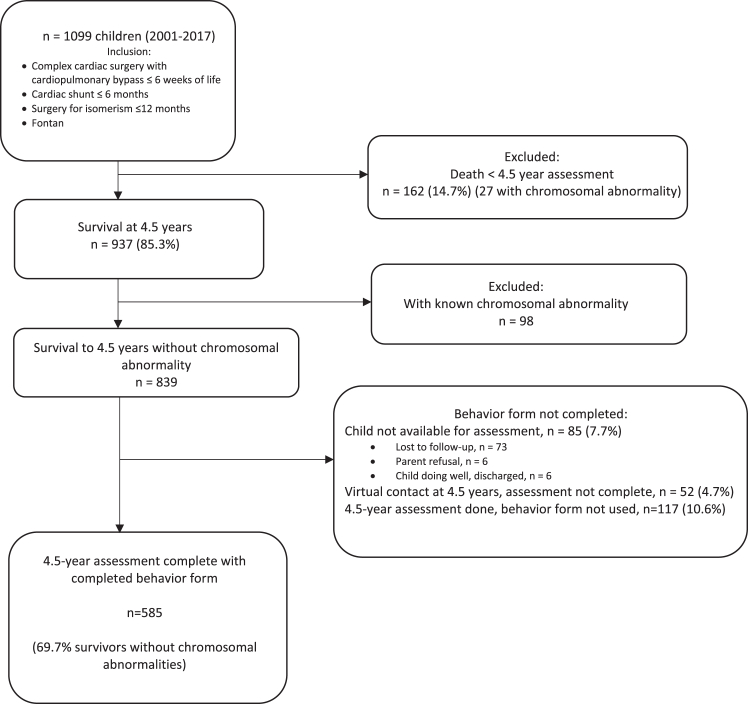
Flowchart describing the study cohort of children after complex cardiac surgery at 4.5 years of age from years 2001 to 2017.

From January 2001 to December 2017, 1099 infants of ≤6 weeks of age had their first CCSx requiring cardiopulmonary bypass, cardiac shunt at 6 months of age including Glenn surgery, or surgery for isomerisms at <12 months of age (Fig. 1).

There were no statistically significant differences in predictor variables between the 585 children with complete behavioural data who received assessment and the 117 (13.9%) children who were assessed and did not have complete behaviour data (Table 1). The children who were lost to follow-up had fewer complications overall (Table 1). Acute care predictors and childhood health variables are also included in Table 1.

**Table 1:** Descriptive acute care and health variables of the study population

Variables	Assessment done with behaviour data (N = 585)	Assessment done, no behaviour data (N = 117)	Virtual contact at 4.5 years, no behaviour data (N = 52)	No behaviour data at 4.5 years (N = 85)	*F/*χ^2^ test	*P* value
At first surgery						
Birth year	2009.1 (4.4)	2009.4 (5.2)	2015.9 (2.2)	2011.7 (3.8)	46.1	<0.001
Gestational age (wk)	38.6 (2.0)	38.3 (2)	37.9 (2.1)	38.3 (1.9)	2.249	0.081
Birth weight (g)	3229.8 (627.1)	3168.3 (639.3)	3190.5 (678.3)	3217.8 (628.3)	0.341	0.796
Sex, female	230 (39.3%)	47 (40.2%)	19 (36.5%)	33 (37.1%)	0.362	0.948
Antenatal diagnosis, no	292 (49.9%)	56 (47.9%)	34 (65.4%)	42 (50%)	5.02	0.170
Surgical severity, complexity, and major complications occurring at anytime up to 4.5 years						
Single ventricle, yes	232 (39.7%)	45 (38.5 %)	28 (53.8%)	24 (27%)	10.35	0.016
Total cardiopulmonary bypass time (min)	165.2 (100)	159.5 (96.9)	192.1 (127.9)	152.9 (82.2)	3.922	0.009
Deep hypothermic circulatory arrest, anytime	348 (59.5%)	60 (51.3%)	24 (46.2%)	37 (43.5%)	11.878	0.008
Convulsions, yes	53 (9.1%)	9 (7.7%)	1 (1.9%)	1 (1.1%)	10.276	0.016
Dialysis, yes	69 (11.8%)	16 (13.7%)	5 (9.6%)	3 (3.5%)	7.740	0.052
Cardiopulmonary resuscitation, yes	44 (7.5%)	14 (12%)	3 (5.8%)	5 (5.9%)	0.228	0.973
Sepsis∗, yes	69 (11.8%)	17 (14.5%)	6 (11.5%)	5 (5.7%)	2.736	0.434
One or more of convulsions, dialysis, cardiopulmonary resuscitation, yes	178 (30.4%)	40 (34.2%)	12 (23.1%)	12 (14.1%)	8.502	0.037
Extracorporeal membrane oxygenation, yes	37 (6.3%)	11 (9.4%)	6 (11.5%)	1 (1.2%)	4.710	0.194
Ventricular assist device, yes	5 (0.9%)	4 (3.4%)	1 (1.9%)	0 (0%)	2.750	0.432
Heart transplantation, yes	20 (3.4%)	7 (6%)	1 (1.9%)	0 (0%)	4.247	0.236
Life-saving support (1 or more of extracorporeal membrane oxygenation, ventricular assistive device, and heart transplantation), yes	51 (8.7%)	15 (12.8%)	6 (11.5%)	1 (1.7%)	7.928	0.048
Total hospitalization (d) (at surgical site)	37.1 (36.6)	39.7 (31.1)	37.3 (33.5)	32.31 (29.2)	0.230	0.795
Total ventilation time (d) (at surgical site)	14.8 (14.3)	12.7 (9.7)	10.0 (9.9)	8.49 (6.2)	5.377	0.001
4.5 years, demographic						
Socioeconomic index, family†	44.6 (13.7)	42.8 (12.8)	–	–	1.376	0.170
Mother’s schooling (y)	13.7 (2.7)	13.3 (2.2)	–	–	1.786	0.076
4.5 years, assessment			–	–		
Height, *Z*-score	–0.4398 (1.48)	–0.3187 (2.07)	–	–	–0.749	0.454
Weight, *Z*-score	–0.3528 (1.19)	–0.2105 (1.22)	–	–	–0.349	0.727
Head circumference, *Z*-score	–0.0623 (1.32)	–0.2457 (1.33)	–	–	1.370	0.171
Gastrostomy, anytime at or before 4.5 years	21 (17.9%)	136 (23.2%)	–	–	1.577	0.209
Number of hospitalizations for noncardiac reasons after first discharge, n	1.50 (2.69)	1.49 (2.65)	–	–	0.025	0.980
Number of hospitalizations for cardiac reasons after first discharge, n	1.77 (2.18)	1.44 (1.91)	–	–	1.665	0.098
Number of medical specialists at 4.5 years, n	2.25 (1.66)	2.16 (1.65)	–	–	0.550	0.582
Pulmonary medications used at 4.5 years	68 (11.6%)	17 (14.5%)	–	–	0.774	0.379
Cardiac medications used at 4.5 years	275 (47%)	56 (47.9%)	–	–	0.029	0.866

Data are presented as mean (SD) or n (%).

∗ Sidhu et al.^18^

† Blishen et al.^17^

### Parent ratings of preschool CCSx survivors

Table 2 shows the number of parents identifying at-risk and clinically significant behaviours in their child for each BASC scale. Of the parents, 29.6% indicated that their child had at-risk or clinically significant BC relating to activities of daily living, 27.2% with somatization, and 25.6% with functional communication.

**Table 2:** Individual scale results for the Behaviour Assessment System for Children for 585 preschool children without known chromosomal abnormalities after early complex cardiac surgery with percent of behaviour concern

Scales	Total cohort, mean (SD)	Behavioural concern, n (%)
Activities of daily living	46.1 (11.8)	173 (29.6)
Somatization	54.4 (11.3)	159 (27.2)
Functional communication	44.6 (10.4)	150 (25.6)
Adaptive skills	46.9 (10.9)	146 (25)
Atypicality	52.8 (11.4)	130 (22.2)
Attention problems	51.4 (9.8)	129 (22.1)
Adaptability	50.3 (10.6)	113 (19.3)
Internalization problems	51.2 (9.8)	111 (19)
Hyperactivity	51.6 (10.1)	110 (18.8)
Behavioural Symptoms Index	51.3 (10.1)	109 (18.6)
Social skills	49.0 (10.2)	107 (18.3)
Withdrawal	50.2 (10.6)	100 (17.1)
Externalization problems	51.2 (9.8)	97 (16.6)
Depression	49.9 (10.5)	94 (16.1)
Aggression	50.6 (9.7)	81 (13.8)
Anxiety	48.4 (9.4)	69 (11.8)

SD, standard deviation.

### Differences in cardiac anatomy

**Figure 2:**
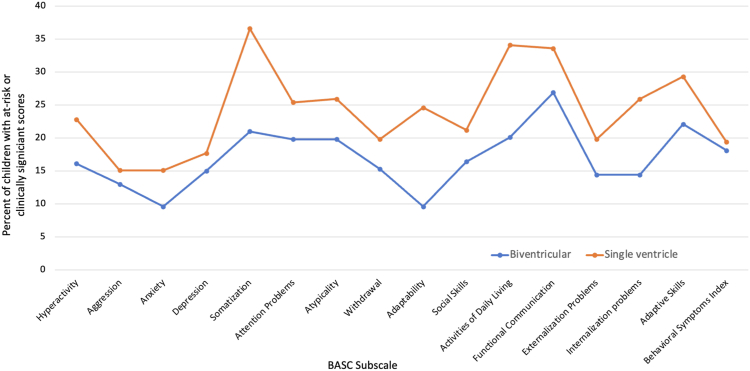
Percent of children with biventricular and single ventricle anatomy with at-risk or clinically significant BASC subscales. BASC, Behaviour Assessment System for Children.

There were 232 of 585 (40%) children with single ventricle anatomy. Figure 2 shows behavioural profiles of children with single and biventricular anatomy. The children with single ventricle anatomy were found to be more likely to have elevations in the at-risk or higher range on the somatization (*P* < 0.001), activities of daily living (*P* < 0.001), adaptability (*P* = 0.01), internalization problems (*P* = 0.001), and anxiety (*P* = 0.05) scales.

### Prediction of behavioural concerns

Univariate and purposeful selection multiple logistic regression analyses are shown in Table 3. Independent predictors of BC were noncardiac hospitalizations (OR: 1.10, 95% CI: 1.02, 1.19; *P* = 0.015) for externalizing behaviours. Noncardiac hospitalizations (OR: 1.14, 95% CI: 1.05, 1.24; *P* = 0.003), female sex (OR: 1.62, 95% CI: 1.04, 2.52; *P* = 0.031), and single ventricle anatomy (OR: 1.82, 95% CI: 1.04, 3.17; *P* = 0.035) were independent predictors for internalizing BC. Noncardiac hospitalizations (OR: 1.10, 95% CI: 1.02, 1.19; *P* = 0.017), SES of the family (OR: 0.98, 95% CI: 0.96, 0.10; *P* = 0.031), and years of maternal schooling (OR: 0.91, 95% CI: 0.84, 0.10; *P* = 0.04) were independent predictors of adaptive BC. Life-saving support (OR: 2.03, 95% CI: 1.01, 3.96; *P* = 0.041) was an independent predictor for BSI, indicating more pervasive behaviours.

**Table 3:** Univariate and multivariate logistic regression to predict the scales of the Behaviour Assessment System for Children-II and -III after early complex cardiac surgery

Variable	Univariate regressions, OR (95% CI)	*P* value	Multivariate regressions, OR (95% CI)	*P* value
Externalizing				
Number of hospitalizations for noncardiac reasons after first discharge, n	1.15 (1.07, 1.24)	<0.001	1.101 (1.022, 1.194)	0.015
Number of medical specialists at 4.5 years, n	1.21 (1.08, 1.36)	0.001		
Extracorporeal membrane oxygenation, anytime to 4.5 years	2.61 (1.23, 5.31)	0.009		
Life-saving support (1 or more of extracorporeal membrane oxygenation, ventricular assist device, and heart transplantation), yes	2.3 (1.17, 4.31)	0.012		
Cardiac medications used at 4.5 years, yes	1.68 (1.08, 2.62)	0.021		
Height at 4.5 years, *Z*-score	0.83 (0.71, 0.98)	0.027		
Cardiopulmonary resuscitation, yes	2.01 (0.96, 3.97)	0.051		
Total cardiopulmonary bypass time (min)	1.002 (0.999, 1.004)	0.055		
Mother’s schooling (y)	0.93 (0.85, 1.01)	0.072		
Single ventricle anatomy, yes	1.46 (1.94, 2.27)	0.088		
Internalizing				
Number of hospitalizations for noncardiac reasons after first discharge, n	1.19 (1.1, 1.29)	<0.001	1.137 (1.051, 1.242)	0.003
Number of medical specialists at 4.5 years, n	1.25 (1.12, 1.4)	<0.001		
Single ventricle anatomy, yes	2.07 (1.36, 3.14)	0.001	1.819 (1.044, 3.174)	0.035
Gastrostomy, anytime at or before 4.5 years	2.11 (1.34, 3.29)	0.001		
Heart transplantation, yes	4.59 (1.84, 11.48)	0.001		
Sex, female	1.76 (1.16, 2.67)	0.008	1.623 (1.044, 2.52)	0.031
Total cardiopulmonary bypass time (min)	1.003 (1.0006, 1.004)	0.01		
Life-saving support (1 or more of extracorporeal membrane oxygenation, ventricular assist device, and heart transplantation), yes	2.11 (1.1, 3.92)	0.02		
Ventricular assist device, yes	6.56 (1.07, 50.22)	0.041		
Birth weight (g)	0.9996 (0.999, 1.00)	0.058		
Hospitalizations for cardiac reasons after first discharge, n	1.09 (0.99, 1.19)	0.06		
Cardiac medications used at 4.5 years, yes	1.48 (0.98, 2.25)	0.063		
Cardiopulmonary resuscitation, yes	1.9 (0.93, 3.68)	0.067		
Antenatal diagnosis, no	1.47 (0.97, 2.24)	0.071		
Adaptive				
Number of hospitalizations for noncardiac reasons after first discharge, n	1.18 (1.1, 1.28)	<0.001	1.098 (1.021, 1.193)	0.017
Number of medical specialists at 4.5 years, n	1.23 (1.1, 1.36)	<0.001		
Gastrostomy, anytime at or before 4.5 years	2.34 (1.54, 3.53)	<0.001		
Life-saving support (1 or more of extracorporeal membrane oxygenation, ventricular assist device, and heart transplantation), yes	3 (1.66, 5.4)	<0.001		
Socioeconomic index, family∗	0.97 (0.96, 0.99)	0.001	0.981 (0.964, 0.998)	0.031
Mother’s schooling, years	0.89 (0.82, 0.95)	0.001	0.914 (0.838, 0.995)	0.04
Total hospitalization, days to 4.5 years (at surgical site)	1.008 (1.003, 1.01)	0.001		
Total ventilation time, days to 4.5 years (at surgical site)	1.02 (1.01, 1.03)	0.001		
Height, *Z*-score at 4.5 years	0.81 (0.7, 0.93)	0.003		
Extracorporeal membrane oxygenation, anytime to 4.5 years	2.76 (1.39, 5.43)	0.003		
Dialysis, anytime to 4.5 years	2.14 (1.26, 3.61)	0.004		
Cardiopulmonary resuscitation, yes	44 (7.5%)	0.005		
One or more of convulsions, dialysis, cardiopulmonary resuscitation, yes	178 (30.4%)	0.005		
Total cardiopulmonary bypass time (min)	1.002 (1.002, 1.004)	0.026		
Cardiac medications used at 4.5 years, yes	1.52 (1.04, 2.21)	0.03		
Heart transplantation, yes	2.56 (1.01, 6.3)	0.041		
Single ventricle anatomy, yes	1.46 (1, 2.13)	0.049		
Gestational age (wk)	0.92 (0.84, 1)	0.051		
Sex, female		0.061		
Birth weight (g)	0.999 (0.999, 1)	0.067		
Ventricular assist device, yes	4.58 (0.75, 35.06)	0.097		
Behavioural Symptoms Index				
Life-saving support (1 or more of extracorporeal membrane oxygenation, ventricular assist device, and heart transplantation), yes	2.66 (1.41, 4.87)	0.002	2.03 (1.013, 3.963)	0.041
Number of medical specialists at 4.5 years, n	1.19 (1.06, 1.33)	0.002		
Number of hospitalizations for noncardiac reasons after first discharge, n	1.09 (1.02, 1.18)	0.009		
Extracorporeal membrane oxygenation, anytime to 4.5 years	2.55 (1.22, 5.11)	0.01		
Ventricular assist device, yes	6.71 (1.1, 51.4)	0.038		
Cardiopulmonary resuscitation, yes	1.94 (0.95, 3.78)	0.057		
Socioeconomic index, family∗	0.99 (0.97, 1)	0.071		
Total ventilation time, days to 4.5 years (at surgical site)	1.01 (0.998, 1.02)	0.091		

CI, confidence interval; OR, odds ratio.

∗ Sidhu et al.^18^

Further analysis identified that children who received life-saving support had 1.091 times higher odds (95% CI: 1.012, 1.176; *P* = 0.024) of experiencing more noncardiac hospitalizations after first surgery compared with children who did not receive life-saving support. Children who had single ventricle anatomy had 1.114 times higher odds (95% CI: 1.036, 1.197; *P* = 0.003) of noncardiac hospitalizations after first surgery compared with children with biventricular anatomy.

## Discussion

This inception-cohort study was restricted to high-risk children who had CCSx in early infancy and examined prospectively the collected risk factors for adverse behavioural outcomes at 4.5 years. Using this design, we presented relevant findings for teams providing ongoing medical, developmental, and rehabilitative services to survivors of CHD. The findings are consistent with previous reports of increased BC in this population. The findings contribute to an existing understanding of behavioural outcomes in survivors of CCSx by identifying independent predictors of BC. Scores of at-risk and clinically significant behaviours were used to define BC, rather than elevated mean scores, as individuals with these cutoff scores would be clinically identifiable for support and intervention. This study also used the BASC, 2nd and 3rd editions,^15^^,^^16^ which, to our knowledge, has not been reported in the literature to date for this population of patients.

### Predicting risk for behavioural concerns: number of noncardiac hospitalizations

The number of noncardiac hospitalizations (NNH) was found to be a risk factor of greater externalizing, internalizing, and adaptive BC. The NNH was an independent predictor on multivariate regressions and not due to single ventricle or life-saving interventions. This finding requires further attention.

Hospitalization, alone, in children has been reported to be associated with higher rates of anxiety and impacts on mental health. It is possible that children with recurrent hospitalizations experienced increased isolation from peers due to presenting health issues, adversely affecting behaviour. Recurrent hospitalizations could also reflect poorer access to primary care to support ongoing optimization of the child’s health in the community. Differential access to primary care was not captured in our study. Recurrent admissions may result in poorer emotional coping in the child.^19^ Younger children under the age of 4 years have also been reported to show more adverse behaviour after hospitalization compared with older children, in particular those who experience social disadvantage.^20^ It is not consistently understood whether this increase in behavioural difficulty is sustained.^21^

The parenting experience of recurrent hospitalizations should also be considered. Our behavioural scale is reliant on parent responses that are impacted by parents’ interpretation of their child’s behaviour. The parents’ relationship with their child and the parents’ own mental health can impact perceptions of child behaviour.^22^ Parents experience psychological distress when their child is acutely hospitalized, and this distress can impact mental health and development of their child.^23^ Recurrent hospitalizations may affect parents’ access to quality childcare, which was not recorded in our study. Further understanding of the parents’ and the child’s experience with recurrent hospitalization is warranted as both experiences can influence the reporting of increased BC.

Of interest, the number of cardiac hospitalizations and total cardiac hospitalization days to age 4.5 years were not independently associated with behaviour outcomes. This suggested that it was noncardiac hospitalizations that were most strongly associated with adverse outcomes for BC. Noncardiac hospitalizations may be more stressful for parents as they are often unexpected and may increase the perception of vulnerability. These admissions were due to illness including viral respiratory infections, asthma, gastroenteritis, seizures, gastrostomy, and injury. It is possible that decisions for noncardiac hospitalizations may have been influenced by the underlying cardiac condition. Efforts to address BC should target both parent and child coping associated with the stress of recurrent noncardiac hospitalization and include efforts to optimize the child’s cardiac and general health in the outpatient setting through access to comprehensive primary care to prevent noncardiac hospitalizations after CCSx.

### Predicting risk for behavioural concerns: life-saving support

Receiving life-saving support such as extracorporeal membrane oxygenation, heart transplantation, or ventricular assist device was an independent predictor of elevated BSI. This index score is indicative of more pervasive behavioural or emotional challenges.^15^ This suggests that the use of life-saving support is an independent risk factor for more severe presentations of emotional or BC.

In our clinical experience, compared with the discrete time point of a surgical procedure, having a child receiving a form of life-saving support is associated with a more stressful experience due to the degree of uncertainty of its duration, course, and outcome. We propose that the family experience of receiving life-saving supports is unique compared with other interventions due to the degree and duration of stress and uncertainty. This can impact the experience of the parent, which can further impact the parent-child relationship and subsequently the behavioural management strategies practiced within the family unit. This finding may be suggestive of a target population to prioritize trauma-informed and trauma attachment–based strategies to address this risk. This hypothesis requires further evaluation.

### Single ventricle or biventricular

A cumulative effect of having single ventricle CHD may be found in comparing behavioural outcomes in the single ventricle and biventricular groups. Both groups were similar in the distribution of their behavioural profiles except that parents of children with single ventricle anatomy reported more elevated reports of at-risk and clinically significant behaviours due to somatization (*P* < 0.001), activities of daily living (*P* < 0.001), adaptability (*P* = 0.01), internalization problems (*P* = 0.001), and anxiety (*P* = 0.05). Children with single ventricle anatomy may be at increased risk of BC compared with children with biventricular anatomy. These children are also at increased risk of BC due to the increased likelihood of recurrent noncardiac hospitalizations as we found.

### Elevated BASC subscales in survivors of CCSx

Particular BASC subscales also require further attention in this population. In particular, the subscales for somatization and activities of daily living require further attention in this population.

### Somatization

Somatization concern was more elevated in the single ventricle population than the biventricular population. Overall, it was also the most elevated subscale across all survivors. Somatization describes an expression of physical distress or symptoms that is inconsistent with medical history, physical examination, or laboratory or imaging investigations (ie, not due to structural organ pathology).^24^ Understanding the predisposing, precipitating, and perpetuating factors is important, as these genuinely experienced symptoms can be debilitating.^25^

Somatization may be influenced by mental health factors such as anxiety in the child or parent, responses to trauma, child temperament, and the child’s previous experiences with pain, and may be associated with attachment issues in the child-parent relationship.^26^, ^27^, ^28^, ^29^, ^30^ Reports of frequent somatization in preschool children have been as high as 15%-20% in the general population.^31^^,^^32^

Somatization in survivors of CCSx is complex because there may be medical factors contributing to physical complaints that could confound parent reporting of these symptoms. There are also risk factors inherent in the child’s medical course that increase the risk of reports of somatization including the impacts of parental stress and child emotional distress and trauma associated with recurrent medical check-ups, major surgeries, and invasive procedures.^33^

Understanding the factors contributing to increased parent reports of somatization in preschool survivors of CCSx is an area that requires further research as addressing these issues may provide strategies for behavioural intervention and improve quality of life.

### Activities of daily living

Decreased functional abilities and lower adaptive daily living skills have been previously reported in children with complex congenital cardiac disease.^34^^,^^35^ Our study identified that children with mothers with less years of education and those from lower SES were at greater risk of BC associated with adaptive daily living. Higher maternal education has previously been identified as one of the most important predictors of a child’s ability to perform everyday adaptive daily activities as expected for their age and stage of development in paediatric survivors of CCSx.^36^ Lower SES has also been found to be associated with poorer neurodevelopmental outcomes including in communication skills and adaptive behaviour.^37^

There are ongoing innovations and system changes aimed at mitigating health disparities and building strategies that prioritize the improvement of equity for patients with congenital heart disease.^38^^,^^39^ Our findings support prioritizing the provision of developmental services to children and families with lower maternal education and lower SES given a higher risk for BC in this population.

### Strengths and limitations

The strengths of this study include the high proportion of children assessed in our developmental follow-up clinics at 4.5 years of age with complete parent rating scales. The prospective design, prespecified clinically relevant potential predictor variables, and relatively large sample size are also strengths. Our study of BC after CCSx contributes to the body of existing literature describing variables impacting behavioural outcomes in children in other contexts, such as those with recurrent hospitalization and those who have experienced stress and adversity due to chronic medical illness.^40^ Trauma-informed individual and family strategies proposed to address BC in other paediatric groups with chronic complex medial needs may be considered as intervention strategies to support survivors of CCSx.^41^, ^42^, ^43^, ^44^, ^45^

The main limitations of the study are that the behavioural scales used are parent-reported. Parental stress, socioeconomic background, which parent or caregiver completes the questionnaire, parent educational level, parent mental health, and child gender are factors that can impact parent reporting of behavioural issues in children.^46^, ^47^, ^48^ This study also does not capture the variability in access to community providers of early intervention supports such as physical therapy, occupational therapy, speech language therapy, and psychology or the variability in service navigation required to access these supports. Nevertheless, as part of the CPTFP clinics, these services are facilitated when thought necessary. Some variables that may be predictive of adverse outcome were not available and included acquired brain injury on brain imaging. At our centre, brain imaging was not routinely performed on survivors of CCSx, and the incidence of stroke was unknown. Future study of this risk factor will be very important.

Another limitation was that this represents a single centre of cardiac surgery, which may limit generalization of our results. Finally, as in all observational studies, the associations reported may not be cause-effect relationships, as there may have been unmeasured confounding variables.

## Conclusions

This study highlights that the NNH after CCSx is a potentially modifiable predictor of increased at-risk and clinically significant reports of internalizing, externalizing, and adaptive behavioural differences. We also identified that children of mothers with lower SES and lower education are especially at risk of increased BC due to adaptive functioning. In addition, children who have received life-saving therapies have increased risk of more pervasive BC. These may represent groups that may benefit from more targeted support. Improving inpatient trauma-informed care experiences and optimizing access to primary care to minimize rates of noncardiac hospitalization may be modifiable approaches that require further attention.
